# Can environmental constraints determine random patterns of plant species co-occurrence?

**DOI:** 10.1002/ece3.1349

**Published:** 2015-02-13

**Authors:** Gonzalo García-Baquero, Rosa M Crujeiras

**Affiliations:** 1 Department of Plant Biology and Ecology, University of the Basque CountryBilbao, Spain; 2 Department of Statistics and Operational Research, University of Santiago de CompostelaSantiago de Compostela, Spain

**Keywords:** Assembly processes, distance-based redundancy analysis, logistic regression, null model analysis, simulation, species co-occurrence

## Abstract

Plant community ecologists use the null model approach to infer assembly processes from observed patterns of species co-occurrence. In about a third of published studies, the null hypothesis of random assembly cannot be rejected. When this occurs, plant ecologists interpret that the observed random pattern is not environmentally constrained – but probably generated by stochastic processes. The null model approach (using the *C*-score and the discrepancy index) was used to test for random assembly under two simulation algorithms. Logistic regression, distance-based redundancy analysis, and constrained ordination were used to test for environmental determinism (species segregation along environmental gradients or turnover and species aggregation). This article introduces an environmentally determined community of alpine hydrophytes that presents itself as randomly assembled. The pathway through which the random pattern arises in this community is suggested to be as follows: Two simultaneous environmental processes, one leading to species aggregation and the other leading to species segregation, concurrently generate the observed pattern, which results to be neither aggregated nor segregated – but *random*. A simulation study supports this suggestion. Although apparently simple, the null model approach seems to assume that a single ecological factor prevails or that if several factors decisively influence the community, then they all exert their influence in the same direction, generating either aggregation or segregation. As these assumptions are unlikely to hold in most cases and assembly processes cannot be inferred from random patterns, we would like to propose plant ecologists to investigate specifically the ecological processes responsible for observed random patterns, instead of trying to infer processes from patterns.

## Introduction

To answer the fundamental question of how species assemble to form communities, plant ecologists often use the null model approach (Götzenberger et al. 2012) introduced by Connor and Simberloff (1979). Assuming that assembly processes can be inferred from observed patterns of species co-occurrence (Harvey et al. 1983), the ecologist tests the null hypothesis of random species co-occurrence (or random assembly). This null hypothesis states how a community would present itself if it were structured only by stochastic factors (Gotelli and Ulrich 2012), that is, in the absence of biotic interactions, dispersal and environmental variability (Götzenberger et al. 2012). Once the hypothesis test is completed, the ecologist draws inferences on the assembly processes that shaped the observed pattern of species co-occurrence (Gotelli and Ulrich 2012).

The null hypothesis of random assembly cannot be rejected in about a third of published experimental plant matrices (Ulrich and Gotelli 2013, Table 7) or in about 60% of published co-occurrence tests (Götzenberger et al. 2012; Table 2). When this occurs, plant ecologists do not use to investigate further the processes responsible for the random patterns. Instead, they may deny a strong influence of deterministic processes (Burns 2007) or suggest that chance (Wilson et al. 1992), lack of equilibrium (Wilson 1988) or dispersal constraints (Reitalu et al. 2008) cause random co-occurrence. They may also discuss the hypothetical existence of combinations of biotic interactions, periodical disturbances and dispersal constraints (Borcard et al. 1992) or unspecified but otherwise nondominant processes (Zhang et al. 2009) that counteract competition. Plant ecologists, in sum, explicitly or implicitly assume that if a plant assemblage presents a random structure, then it is not environmentally determined – but instead is caused by stochastic processes.

For that reason, when we found that an undisturbed and environmentally driven assemblage of alpine hydrophytes in Iberian soft water lakes presented itself as randomly structured, it seemed to be a theoretical contradiction. However, the mistake – if existing – was not evident. On the one hand, our null model analysis appeared correct. On the other hand, our ongoing study agreed with prior research, which shows how species composition in Pyrenean (Gacia et al. 1994) and northern European (Murphy 2002) soft water lakes is explained by physical constraints (lake area and elevation) and trophic state (water conductivity and pH). These facts have been used in lake restoration (Brandrud 2002; Brouwer et al. 2002), and, crucially, the causal links between hydrophyte presence–absence and trophic state at a finer scale are known. Trophic state is mostly determined by catchment characteristics (Brönmark and Hansson 2005), which, in alpine systems, are bedrock and surrounding vegetation (Catalan et al. 1993). Conductivity is a proxy for cations (Gorham et al. 1983), which are limiting in soft water lakes, so nutrient enrichment enhances the growth of, for instance, *Ranunculus peltatus* Schrank (Roelofs 1983). Change in pH is related to the carbon dioxide-bicarbonate system (Brönmark and Hansson 2005), and the emergence of *Sparganium angustifolium* Michx and other species on limed lakes is related to increased availability of inorganic carbon (Brandrud 2002; Lucassen et al. 2009).

Hence, aiming to solve this apparent theoretical contradiction, we considered a first question: Can environmental constraints determine random patterns of plant species co-occurrence? An affirmative answer would lead to theoretical implications, but a mere demonstration that environmental determinism and random pattern co-occur would not suffice. A satisfactory explanation should also reveal the precise pathway between environmental constraints and species co-occurrence (Cox and Donnelly 2011). This led us to ask a second question: How do environmental constraints generate a random pattern?

## Material and Methods

### Field survey and study area

The presence–absence of aquatic vascular plants (“hydrophytes”) in the *n *=* *17 permanent lakes that exist in the Gredos Massif (Central System, Spain) was surveyed by means of line transect sampling (Krebs 1999). In each lake or pond, line transects of width 1 m were laid out in a radial pattern and searched for plant species. The lake shoreline constituted the baseline along which the beginning of each transect was randomly located; the end of each transect was the geometric center of the lake. The number of transects used for each lake was not fixed in advance. Instead, in order to minimize sampling effort and yet achieve the same level of precision for all lakes, sequential sampling (Thompson 1992; Krebs 1999) was used. An initial random sample of five transects was selected for each lake. Additional transects (also randomly selected) from the same lake were added to the sample using the decision (stopping) rule to quit sampling as soon as three subsequent transects added no new species to the lake species list. The lakes occur in three sectors (Bejar, E Gredos and W Gredos), and the aquatic vegetation is classified as *Littorellion uniflorae* Koch 1926 (Sardinero 2004). According to prior research (Catalan et al. 1993; Gacia et al. 1994), we measured conductivity (expressed as specific conductance in *μ*S cm^−1^ at 25°C) and pH in water samples once (July 2008). Conductivity and pH were measured in each of the five transects that compose the above-mentioned initial sample, and the values here reported are lake averages. Lake area and elevation data were obtained from Toro et al. (2006). The vegetation surrounding the lakes consists of *Nardus* and *Festuca* pastures and *Juniperus*-*Cytisus* scrubs. The bedrocks are formed by mineralogically complex and broadly variable monzogranites and granodiorites (Gibbons and Moreno 2002).

### Data analysis

A three-matrix dataset was constructed: a species composition matrix of 17 lakes × 9 hydrophytes; a geographic matrix of 17 lakes × 2 Cartesian coordinates (*X*,*Y*) derived from latitude and longitude; and an environment matrix of 17 lakes × 2 physical descriptors (elevation in m and lake area in m^2^) and two chemical descriptors (pH and water conductivity in *μ*S cm^−1^). The geographic matrix is necessary to test for spatial autocorrelation and check the assumptions of independence of errors in the context of logistic and Poisson regression (see below). The physical descriptors were obtained from Toro et al. (2006). The analysis summarized next was carried out using R software v.2.15.2 (R Core Team 2012). Supporting information provides the full dataset (Appendix S1) and R code (Appendix S2) to replicate the analysis described below (section 0 of this code is an exploratory analysis that is not reported in the Results section).

The null hypothesis of random assembly was tested using the *C*-score (Stone and Roberts 1990) and the discrepancy (Brualdi and Sanderson 1999) indices. Their distributions were simulated by 1000 iterations of the sequential swap algorithm (Gotelli and Entsminger 2003), using the function “oecosimu” in the “vegan” package (Oksanen et al. 2010); row and column sums were kept fixed (Gotelli 2000). Thus, one-sample tests were constructed, where the swap algorithm and the fixed-fixed constraints (standard choices to mimic stochastic assembly) create tests with low probability of type I error and good power to detect departures from random assembly (Ulrich and Gotelli 2007, 2013). Benchmark research (Ulrich and Gotelli 2013; Table 4) shows that this type of null model analysis can generally detect 75–80% of departures from random expectation; hence, type II errors are relatively unlikely. The analysis described in this paragraph corresponds to section 1 in the accompanying R code. Additionally, a second test that used the C-score metric and maintained fixed rows and incorporated site (column) weights was performed, where the area of the lakes was used as weights. Row sums were kept fixed to prevent type I errors; weights for the sites (columns) were used because the lakes differ much in size (online resource 1), and hence, they cannot be assumed to be equiprobable. As this type of simulation is not available in the vegan package, *Ecosim* software (Gotelli and Entsminger 2011) was used to carry out this test.

The “vegan” package (Oksanen et al. 2010) was used to test for spatial autocorrelation in species composition and to carry out a partition of variation in community composition according to environmental descriptors (mission V3 in Anderson et al. 2011). In both analyses, the response was a dissimilarity matrix based on the Jaccard coefficient (Legendre and Legendre 1998); a dummy species was previously added (Clarke et al. 2006) to circumvent the double zero problem. To test for spatial autocorrelation in species composition, a Mantel correlogram (Mantel 1967; Oden and Sokal 1986) with Holm correction (Holm 1979) was used to obtain correct *P*-values. To partition variation in community composition according to environmental descriptors, distance-based redundancy analysis (dbRDA) (Legendre and Anderson 1999) was used. dbRDA is an extension of regression to multivariate responses (Legendre and Legendre 1998). Here, dbRDA was implemented via the function “capscale” of “vegan”. Backward and forward selection with the AIC criterion was applied with the function ‘ordistep’ to find a parsimonious dbRDA model. Finally, a posterior partitioning of variation (Borcard et al. 1992; Anderson and Gribble 1998) in species composition between chemical and physical components was carried out. The analysis described in this paragraph corresponds to sections 2 and 3 in the accompanying R code.

Species’ responses were studied using logistic regression (McCullagh and Nelder 1989; Madsen and Thyregod 2010). We tested the dependence of the probability of presence on the environmental gradients for all the species in the dataset, except those (*E. acicularis*,*M. alterniflorum,* and *S. aquatica*) with very low frequency (*n *≤* *2). Model selection with the AICc criterion (Burnham and Anderson 2002) was carried out with the function “dredge” in package “MuMin” (Barton 2012) to find parsimonious models that minimize the loss of information. As AICc does not assess how well a model fits the data, the function “lrm” in the R package “rms” (Harrell 2012) was used to check the fit of logistic models. For each fitted model, an analogue of *R*^2^ was calculated as follows: 1-(null deviance/model deviance). Overdispersion was assessed using the ratio of residual deviance to degrees of freedom. The assumption of independence of errors was tested using spline correlograms (BjØrnstad and Falck 2001). The analysis described in this paragraph corresponds to section 4 in the accompanying R code.

### Simulation of species assembly

Species assembly under two simultaneous processes, one leading to species aggregation and the other leading to species segregation, was simulated 500 times. In the manner of the experimental matrix considered in this work, 60% of species were simulated as constrained by two environmental gradients. For these species, the probability of occurrence depended on the values of two environmental gradients (*X*_1_ and *X*_2_) at each site. Values for *X*_1_ and *X*_2_ were generated from independent normal distributions with mean 2.5 and standard deviation 1.5, in such a way that slightly more than 90% of the possible values lie within the interval [0,5]. The species occurrences in a certain site depended on gradients, *X*_1_ and *X*_2_, by the combination of two probability functions that were either decreasing on *X*_1_ and increasing on *X*_2_ or increasing on both *X*_1_ and *X*_2_. Occurrence probability in each location was modeled through generalized logistic functions, where the product of *f*_1_(*X*_1_) and *f*_2_(*X*_2_) was used as event probability in a Bernoulli experiment. Also in the manner of the experimental matrix considered in this work, the presence/absence in the 25 different sites for the other 40% of species was simulated without reference to any environmental gradient, that is by considering random occurrences with probabilities of either 0.05 or 0.4. In this way, both frequent and infrequent species were modeled.

As a result, we achieved a collection of five hundred 25 × 10 matrices, where each element of each matrix represented the presence/absence of species at a site. These simulated matrices correspond to five hundred scenarios of species assembly under simultaneous processes of species aggregation and segregation. Then, C-Score and discrepancy index tests of the null hypothesis of random assembly were applied to the simulated matrices. These tests were constructed using the swap algorithm and fixed-fixed constraints.

## Results

Nine hydrophytes were found. Six were euhydrophytes *sensu* Den Hartog and Segal (1964): *Callitriche brutia* Petagna (relative frequency = 0.42), *Isoetes velatum* subsp. *asturicense* (M. Laínz) Rivas Mart. & Prada (r. f. = 0.47), *Subularia aquatica* L. (r. f. = 0.12), *Myriophyllum alterniflorum* DC. in Lam. & DC. (r. f. = 0.06), *Ranunculus peltatus* Schrank (r. f. = 0.41), and *Sparganium angustifolium* Michx (r. f. = 0.47). Two were pseudo-hydrophytes*: Antinoria agrostidea* fma. *natans* (Hackel) Ascherson & Graebner, Syn. Mitteleur. Fl. 2(1): 97. 1899 (r. f. = 0.41), and *Juncus bulbosus* var. *fluitans* (Lam.) Beck (r. f. = 0.24). The last one, *Eleocharis acicularis* (L.) Roem. & Schult., was an amphiphyte (r. f. = 0.06). The conductivity values here reported (Table S1 in online resource 1) are very low, though natural in small headwater lakes on igneous rocks.

### The aquatic vegetation of the Gredos lakes presents itself as randomly structured

**Figure 1:**
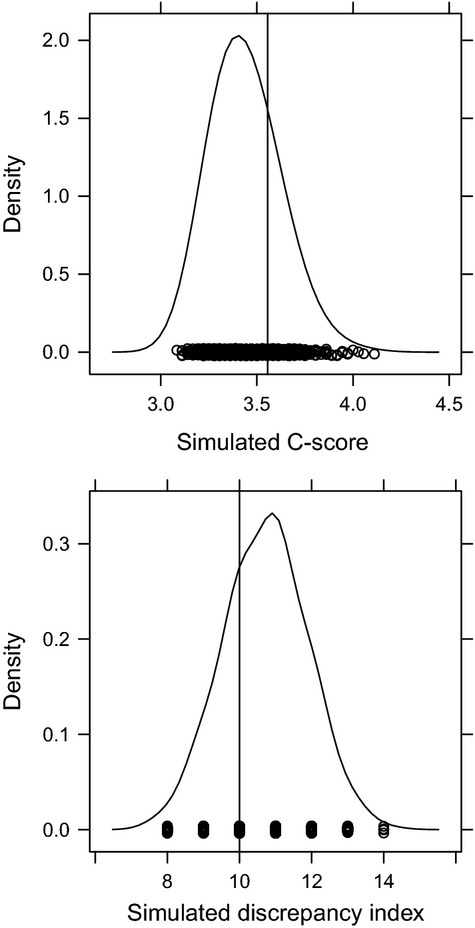
Comparison of the observed indices (vertical lines) to the densities simulated under the null hypothesis of random assembly. The densities (both the *C*-score and the discrepancy index are treated as continuous) were generated using 1000 iterations with the sequential swap algorithm and the fixed-fixed null model. None of the tests were significant.

Both tests (Fig.1) using the fixed-fixed simulation were not significant (observed *C*-score index = 3.6, with mean of simulated indices = 3.4 and *P *=* *0.4; observed discrepancy index = 10.0 with mean of simulated indices = 10.7 and *P *=* *0.8). The test that used the fixed-weighted simulation was not significant either (observed C-score index = 3.6, with mean of simulated indices = 2.7 and variance = 1.0; *P*(observed ≤ expected) = 0.802; *P*(observed ≥ expected) = 0.203). Hence, the null hypothesis of random assembly cannot be rejected.

### The species composition is environmentally determined

**Figure 2:**
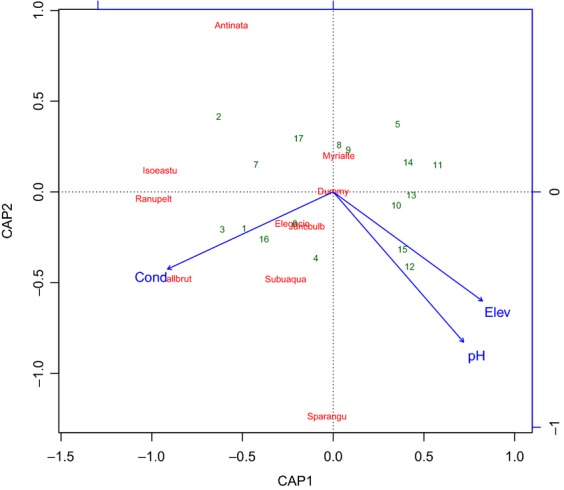
Plot of the distance-based redundancy analysis model summarized in Table2. Numbers identify lakes (1–4: Sector Bejar; 5–8: Sector W Gredos; 9–17: Sector E Gredos). Antinata = *Antinoria natans*; Callbrut = *Callitriche brutia*; Eleoacic = *Eleocharis acicularis*; Isoeastu = *Isoetes asturicense*; Juncbulb = *Juncus bulbosus*; Myrialte = *Myriophyllum alterniflorum*; Ranupelt = *Ranunculus peltatus*; Sparangu = *Sparganium angustifolium*; Subuaqua = *Subularia aquatica*. Elev = elevation; Cond = conductivity; CAP = canonical analysis of principal coordinates.

**Figure 3:**
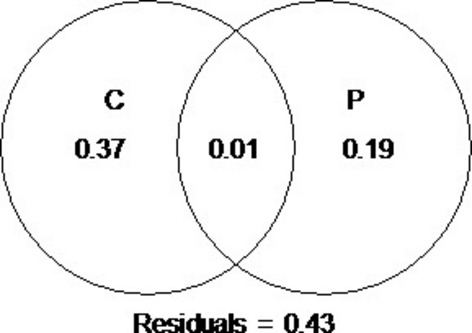
Partitioning of variation in species composition of the aquatic vegetation in the Gredos lakes (Spain) between chemical (C) and physical (P) components after redundancy analysis (Table2 and Fig.2). The chemical component includes pH and conductivity, but the physical component includes only elevation (lake area was not significant).

None of the Mantel statistics were significant (*α *= 5%) after Holm's correction, and hence, the species composition, as measured by the Jaccard coefficient, is not spatially structured at distances 0–20 km. The environmental variables (Table1) pH and conductivity (chemical component) and elevation (physical component) explained *R*^2^ = 57% (*R*^2^–adj. = 47%) of variation in community composition (Table2), but lake area and geographic sector were not significant. In the corresponding constrained ordination (Fig.2), the first axis (CAP1) is strongly related with conductivity, whereas the second axis (CAP2) is mainly related with pH and secondarily with elevation. Partitioning of variation (Fig.3) shows that the vegetation, as expected, is primarily structured by the pure chemical component (*R*^2^ = 37%), with a quantitatively less important contribution by the pure physical component (*R*^2^ = 19%). The very small overlap between both components (*R*^2^ = 1%) occurs because pH is partly structured through elevation, with the higher lakes possessing more neutral conditions than the lower lakes – which are slightly more acidic.

**Table 1:** Descriptive statistics of environmental variables and hydrophyte richness in *n *=* *17 permanent lakes of the Gredos Massif (Central System, Spain). Chemical descriptors (conductivity and pH) were measured in mid-summer. Physical descriptors (elevation and lake area) were obtained from Toro et al. (2006).

Variable	Maximum	Minimum	Range	Mean	SD
Conductivity (*μ*S cm^−1^)	15.4	3.4	12.0	7.0	3.3
pH	7.0	5.8	1.2	6.3	0.3
Elevation (m a.s.l.)	2300	1595	705	2019	168
Lake area (ha)	20.3	0.1	20.2	3.3	5.2
Species Number	7	0	7	3	2

**Table 2:** Parsimonious distance-based redundancy analysis (dbRDA) results (see plots in Figs.4): (i) model summary, (ii) marginal effects of terms, and (iii) variation explained by individual axes. The response is a dissimilarity matrix computed on the presence–absence of hydrophytes in the Gredos lakes (*n *=* *17) using the Jaccard coefficient. Variance inflation factors are 1.52 (conductivity), 1.68 (pH), and 1.24 (elevation).

	df	var.	*F*	*P*
(i)				
Model	3	1.94	5.28	0.001
Residual	13	1.59		
*R*^2^ = 57%				
Adj. *R*^2^ = 47%				
(ii)				
Conductivity	1	0.85	6.97	0.002
pH	1	0.44	3.57	0.008
Elevation	1	0.65	5.31	0.002
Residual	13	1.59		
(iii)				
CAP1	1	1.24	10.13	0.001
CAP2	1	0.54	4.44	0.005
CAP3	1	0.16	1.27	0.271
Residual	13	1.59		

CAP, canonical analysis of principal coordinates axes.

**Figure 4:**
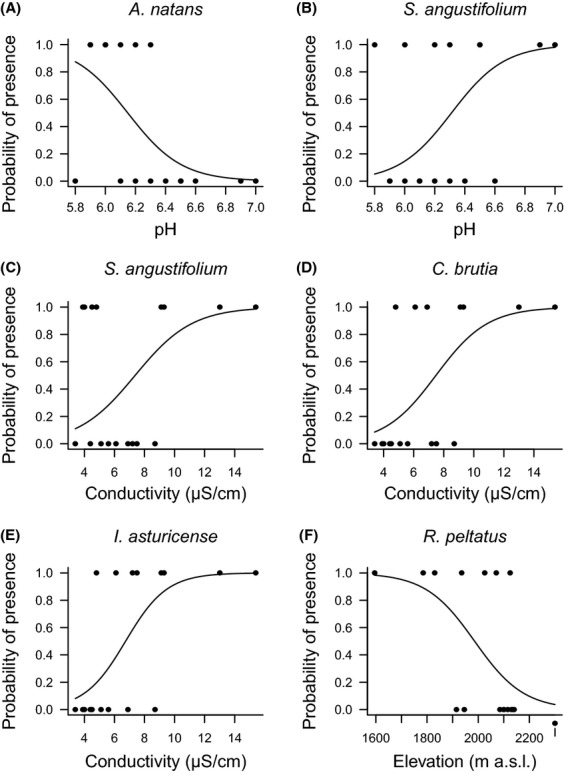
Fitted relationships (parsimonious logistic models) between species *A. natans* (A) *S. angustifolium* (B–C), *C. brutia* (D), *I. asturicense* (E) and *R. peltatus* (F) probability of presence and environmental predictors (see Table3).

Regarding species and the first ordination axis (Fig.2), all of them are positively correlated with conductivity (hydrophytes seem to require minimum conductivity values of about 4–5 *μ*S cm^−1^), but species such as *C. brutia* or *I. asturicense* are strongly correlated (preferring about 8–9 *μ*S cm^−1^). These species appear only in lakes with higher conductivity conditions, but, crucially, they do not exclude less exigent species. This constitutes a process of species aggregation that is constrained by increasing conductivity. This aggregation process is confirmed not only by the species’ individual responses (Table3, Fig.4), but also by the presence of a positive significant association between hydrophyte richness and conductivity revealed by the Spearman rank correlation coefficient (*r*_*S*_ = 0.679, *p *=* *0.003). In relation with the second axis, most species are associated with medium pH conditions. However, as further confirmed by logistic regressions (Table3, Fig.4), *A. natans* appears associated with lower pH conditions, and, in contrast, *S. erectum* is associated with a more neutral pH. This is a process of species turnover that is constrained by pH and elevation gradients.

**Table 3:** Summaries of logistic regressions testing the dependence of the mean probability of presence on environmental gradients for *Antinoria natans* (*R*^2^ = 0.29), *Callitriche brutia* (*R*^2^ = 0.30), *Isoetes asturicense* (*R*^2^ = 0.35), *Ranunculus peltatus* (*R*^2^ = 0.28), and *Sparganium angustifolium* (*R*^2^ = 0.36). No model was fitted for *Juncus bulbosus*. As no overdispersion was found, the dispersion parameter was taken to be 1 in all cases. See plots in Fig.4.

Species	Null deviance	Residual deviance	Parameter	Estimate	SE	*z*	*P*
*A. natans*	23.04	16.41	Intercept	34.25	17.88	1.9	0.06
	on 16 df	on 15 df	pH	−5.57	2.90	−1.9	0.05
*C. brutia*	23.04	16.12	Intercept	−4.35	2.13	−2.0	0.04
	on 16 df	on 15 df	Conductivity	0.58	0.31	1.9	0.06
*I. asturicense*	23.51	15.2	Intercept	−4.91	2.37	−2.1	0.04
	on 16 df	on 15 df	Conductivity	0.73	0.36	2.0	0.04
*R. peltatus*	23.04	16.63	Intercept	20.71	11.07	1.9	0.06
	on 16 df	on 15 df	Elevation	−0.01	0.01	−1.9	0.06
*S. angustifolium*	23.51	15.01	Intercept	−39.42	18.44	−2.1	0.03
	on 16 df	on 14 df	Conductivity	0.53	0.25	2.1	0.03
			pH	5.66	2.75	2.1	0.04

Regarding the sampling units (the lakes), 13 of them (numbered 5–17 in Fig.2), belonging to the sectors E Gredos and W Gredos, are characterized by differing combinations of pH and conductivity levels. The Bejar lakes (1–4 in Fig2), though generally more acidic and with a relatively high conductivity, may also have a relatively high pH (lake 4) and a relatively low conductivity (lake 2). Consistently, lakes 5 and 8 (W Gredos) and lakes 11 and 14 (E Gredos), which are characterized by acidic and low-conductivity conditions, harbor none species or just one. In contrast, lakes with higher conductivity conditions (*e.g.,* 1 in Bejar and 6 in W Gredos) harbor 5–7 hydrophytes. Lake 9 harbors seven species, although it has low conductivity. However, it is one of the larger lakes in Gredos, and, although lake area was not significant in this analysis, this descriptor is a known ecological factor affecting hydrophytes.

### Contrasting processes of species segregation and aggregation seem to counterbalance each other to co-generate a resulting pattern of random species co-occurrence

As shown above, community composition is determined by at least two contrasting processes. The first process, species turnover along pH and elevation gradients, leads to species segregation (turnover). The second process consists of species aggregation constrained by increasing conductivity. The segregation process seems to generate more checkerboards than would be expected by chance (see examples in Table4A–C). In contrast, the aggregation process seems to generate fewer checkerboards than would be expected by chance (see examples in Table4D–F). Overall, these contrasting processes seem to counterbalance each other to co-produce a resulting pattern of species co-occurrence that is not aggregated or segregated, but *random*.

**Table 4:** Examples of species pairs contributing to patterns of segregation (A, *Antinoria natans vs. Sparganium angustifolium*; B, *Callitriche brutia vs. Antinoria natans*; C, *Sparganium angustifolium vs. Ranunculus peltatus*) and aggregation (D, *Callitriche brutia vs. Isoetes asturicense*; E, *Isoetes asturicense* vs. *Ranunculus peltatus*; F, *Ranunculus peltatus vs. Callitriche brutia*). Co-occurrence and checkerboard-like patterns are shaded. The null hypothesis of no more co-occurrence than expected by chance was not rejected for A, B, and C in Pearson's chi-squared tests (*ν *= 1) with Yates’ continuity correction (A: *Χ*^2^ = 0.00, *P *=* *0.999; B: *Χ*^2^ = 0.38, *p *=* *0.536; C: *Χ*^2^ = 0.04, *P *=* *0.839). In contrast, the same null hypothesis was rejected for D, E, and F (D: *Χ*^2^ = 4.74, *P *=* *0.029; E: *Χ*^2^ = 4.74, *P *=* *0.029; F: *Χ*^2^ = 6.87, *P *=* *0.009). The numbers identify the lakes as in Table1 of online resource 1. Lakes 8, 11, and 14 harbor no species.

A	1	9	6	4	2	13	5	15	7	12	17	3	11	10	8	14	16
*A. natans*	1	1	1	0	1	0	1	0	1	0	1	0	0	0	0	0	0
*S. angustifolium*	1	1	1	1	0	1	0	1	0	1	0	1	0	0	0	0	0
B	1	2	9	6	3	5	16	17	4	7	8	10	14	12	11	15	13
*C. brutia*	1	1	1	1	1	0	1	0	1	0	0	0	0	0	0	0	0
*A. natans*	1	1	1	1	0	1	0	1	0	1	0	0	0	0	0	0	0
C	9	6	1	3	15	2	12	16	13	7	4	11	14	5	8	17	10
*S. angustifolium*	1	1	1	1	1	0	1	0	1	0	1	0	0	0	0	0	0
*R. peltatus*	1	1	1	1	0	1	0	1	0	1	0	0	0	0	0	0	0
D	1	2	3	4	6	9	7	16	17	5	8	10	11	12	13	14	15
*C. brutia*	1	1	1	1	1	1	0	1	0	0	0	0	0	0	0	0	0
*I. asturicense*	1	1	1	1	1	1	1	0	1	0	0	0	0	0	0	0	0
E	9	6	1	3	2	7	4	16	17	15	12	13	11	14	5	8	10
*I. asturicense*	1	1	1	1	1	1	1	0	1	0	0	0	0	0	0	0	0
*R. peltatus*	1	1	1	1	1	1	0	1	0	0	0	0	0	0	0	0	0
F	1	2	3	16	9	6	4	7	8	10	11	12	13	14	15	5	17
*R. peltatus*	1	1	1	1	1	1	0	1	0	0	0	0	0	0	0	0	0
*C. brutia*	1	1	1	1	1	1	1	0	0	0	0	0	0	0	0	0	0

### Assembly simulations under simultaneous processes of species aggregation and segregation generate random patterns of species assembly over 90% of times

**Figure 5:**
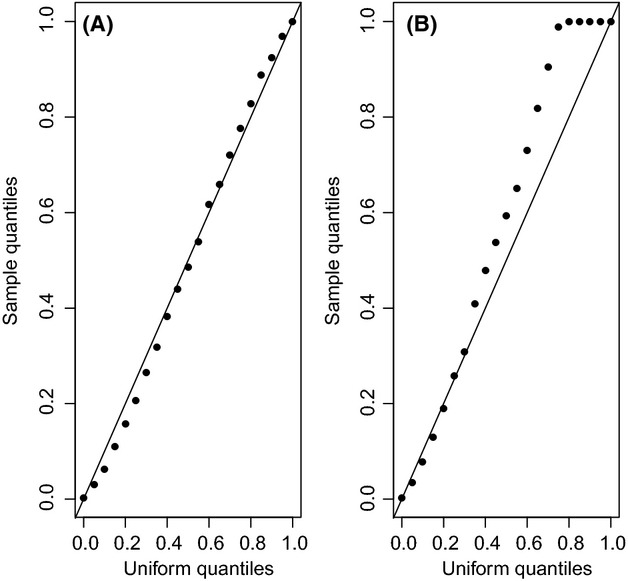
Q-Q (quantile-quantile) plots showing the agreement between the simulation (sample) *P*-value quantiles and the [0,1]-uniform quantiles for (A) the *C*-score test and (B) the discrepancy index test. Under the null hypothesis of random assembly, and assuming that the tests are well calibrated, the dots should be close to the diagonal line.

**Figure 6:**
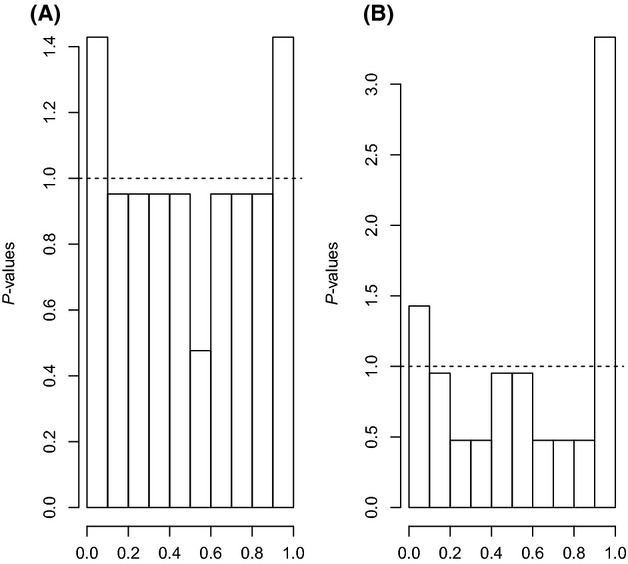
Histograms of simulated *P*-values for (A) the *C*-score and (B) the discrepancy index tests. Under the null hypothesis of random assembly, and assuming that the tests are well calibrated, the *p*-values should follow a uniform distribution in [0,1], and hence, the *p*-value distribution should be close to the horizontal line.

The collection of simulated matrices had, on average, 28% of species presences. The subsequent tests of the null hypothesis of random assembly, at 5% significance level, lead to the rejection of the said null hypothesis 8.4% of the times with the *C*-Score test and 8.6% of the times with the discrepancy index test. In other words, simulation of species assembly under concurrent contrasting processes leading to species aggregation and segregation generated random assembly patterns about 91.5% of times. Although simulation results (Fig.5) suggest that the *C*-score test is better calibrated than the discrepancy test (a property inherent in the tests, not in the simulation), the *p*-value distributions (Fig.6) show that simulation of species assembly under the said contrasting processes generates random patterns most of the time, as expected. Therefore, the simulation results support the suggestion that concurrent processes of species segregation and aggregation counterbalance each other to co-generate a resulting pattern of random species co-occurrence.

## Discussion

### Sources of uncertainty and causality

Readers might object that these results rely on mere relationships found in survey data, not in manipulative research. Hence, to find evidence supporting causality, now we aim to revise the two sources of uncertainty usually present in survey data (Cox and Donnelly 2011). The first is that the ordering of the variables might be wrong. If so, the (supposedly) right ordering would imply the use of the lake environment as response and the hydrophyte presence–absence as explanatory variables. This, however, is contrary to the field knowledge, which affirms that bedrock, soil, and surrounding vegetation shape the chemical composition of the lakes (Catalan et al. 1993; Brönmark and Hansson 2005), not the opposite. Hence, we believe that our ordering of the variables (hydrophyte presence/absence as response and environmental characteristics of the lakes as explanatory variables) is right.

The second source of uncertainty is the (supposed) presence of third variables controlling both response and potential cause. To “help strengthen (…) a causal effect”, Cox and Donnelly (2011) recommend B. Hill's guidelines (Hill 1965). Accordingly, evidence that an association is causal if (1) the association is strong, which in this case is true (Tables2 and 3, Figs.4), and (2) the association has an explanation that is available beforehand, which – as explained in the introduction – is also true (see Roelofs et al. 1984; Brouwer et al. 2002; Murphy 2002; Brönmark and Hansson 2005). Similarly, causality is supported if (3) the effect is found in independent studies, which is certain (Gacia et al. 1994; Murphy 2002), and (4) the association is based on manipulative research. Our results are consistent with prior manipulative research: For example, liming and nutrient enrichment have been shown to cause the emergence of *S. angustifolium* (Lucassen et al. 2009) and *R. peltatus*, (Roelofs 1983), respectively, which are relationships found here (Tables2 and 3; Figs.2 and 4). Likewise, (5) a potential cause must precede its proposed effect, which is also certain because, as earlier explained, variation in pH and conductivity precedes variation in species co-occurrence. Equally (6) monotonic relationships support causality. This is certain because the logistic regressions (Fig.4) show how the presence/absence of species is monotonically explained by pH, conductivity, and elevation. Finally, (7) a causal effect should be specifically generated by a defined pathway. Here, the pathway consists of two contrasting processes that compensate each other to co-generate the observed pattern of co-occurrence, which is not aggregated or segregated, but *random*. In sum, we believe that evidence supports causality.

### What is the underlying mechanism that generates the pattern of random species co-occurrence?

We have shown that community composition in the Gredos lakes is determined by two contrasting processes of species turnover (constrained by pH and elevation) and species aggregation (constrained by increasing conductivity). Also, it has been shown by means of examples that segregation generates more checkerboards than expected by chance and that aggregation generates more co-occurrence than expected by chance. Thus, the observed patterns of segregation and aggregation strongly suggest that these contrasting environmentally constrained processes might counterbalance each other to co-produce a pattern of random species co-occurrence. Although the effect of the aggregation process on community composition seems to be larger than the effect of the turnover process, the resulting effect seems to create a pattern of species co-occurrence in the Gredos lakes that is not aggregated or segregated, but *random*. We believe that our simulation study supports this suggestion.

### What can we infer from a random pattern of species assembly?

Assuming that patterns of species co-occurrence can be used to draw inferences about assembly processes (Harvey et al. 1983; Gotelli and Ulrich 2012), plant ecologists either explicitly or implicitly judge that if an undisturbed plant assemblage presents a random pattern of species co-occurrence, then the pattern is not environmentally determined – but totally or partly caused by stochastic processes (Wilson 1988; Wilson et al. 1992; Burns 2007; Boschilia et al. 2008; Reitalu et al. 2008). However, under the logic of Neyman–Pearson hypothesis testing (Underwood 1997; Lehmann and Romano 2005), which is used in the null model approach (Gotelli and Ulrich 2012), if a null hypothesis is not rejected, the conclusion is that the alternative hypothesis is disproven – but the null hypothesis itself is not proven. Hence, when the null hypothesis of random assembly cannot be rejected, the test conclusion should not be that stochastic processes caused the observed random patterns (which remains unproven), but that biotic interactions, dispersal, and environmental variability – in sum, the ecological processes excluded under the null hypothesis – did not cause the observed random patterns. This conclusion, however, is rarely written down by plant ecologists – though Burns (2007) did. To sum up, when upon retention of the null hypothesis of random assembly, a researcher denies environmental determinism, he or she is inferring a right conclusion – but it cannot imply prevalence of stochastic processes. This is a posterior explanation, not a logical conclusion under Neyman–Pearson hypothesis testing.

Also, this research provides a counter-example where a plant assemblage that presents itself as randomly structured is indeed environmentally determined. It is a single example but, on the one hand, suffices to prove the generality of the assertion that “prevalence of contingent processes can be inferred from random assembly patterns” to be false. On the other hand, environmental determinism (as suggested here) and stochastic processes (presumably but not positively proved), two clearly different processes, might cause the same random pattern. Additionally, Ulrich (2004) has shown that computer-simulated neutral dispersal – a true stochastic process – leads to patterns of segregation, not to random patterns. So, in consequence, we cannot infer stochastic assembly or deny environmental determinism from random patterns. Hence, we believe that, upon retention of the null hypothesis of random assembly (and assuming that a type II error did not occur), we only can conclude that the observed pattern is neither segregated nor segregated, but *random*.

### Are there unstated assumptions in the null model approach?

There remains a paradox to be explained. If denial of environmental determinism is a logical consequence of retaining the null hypothesis of random assembly, but (as suggested here) a random pattern might be caused by environmental constraints… Where is the mistake? We believe that the paradox arises from unstated assumptions of the research model from which the null hypothesis derives. This simple research model, without specializing too much (Gotelli and Ulrich 2012), states that biotic interactions, dispersal and environmental variability structure species co-occurrence (Götzenberger et al. 2012). However, we believe that the model (though apparently simple) assumes that a single ecological factor prevails and structures species co-occurrence or that if several factors co-exist, then all of them act in a unique direction, generating either aggregation or segregation. Consequently, if two opposing processes concurrently constrained a community with contrasting effects, thus co-generating a pattern which would be neither segregated nor segregated (but *random*), then an apparently paradoxical result might occur. Our simulation study supports this suggestion.

The advantages of the simplicity of null model analysis versus more explicit models have been emphasized (Gotelli and Ulrich 2012). However, regarding plant communities, are the unstated assumptions of the implicit research model plausible? We do not reject the idea that these model assumptions are probably tenable in some cases as, for example, relatively simple plant communities inhabiting harsh environments. Nevertheless, given the usual complexity of ecosystems, we believe that these assumptions would be untenable in many other cases. In our case, for example, two ecological processes have been found to explain only about half of the variation in species co-occurrence. Clearly, one or perhaps more (unknown) biotic or abiotic factors might also exert an extra influence in the assembly of the hydrophytes community.

## Conclusion

We have suggested that two environmentally constrained processes of species segregation and aggregation might co-generate the random pattern of hydrophyte co-occurrence found in an Iberian ecosystem of soft water lakes. This apparently paradoxical suggestion has been supported by means of a simulation study. On the other hand, we have also suggested that the null model approach assumes that a single ecological factor prevails or that if several factors decisively influence the community, then they all exert their influence in the same direction, generating either aggregation or segregation. If we are right, these assumptions are unlikely to hold in many cases, and, in consequence, we would like to propose plant ecologists to investigate specifically the ecological processes responsible for observed random patterns, instead of trying to infer processes from patterns.

## Data Accessibility

R scripts and data uploaded as online supporting information.

## Supporting Information

**Table S1.** Location, physical characteristics and mid-summer chemical descriptors of *n* = 17 soft-water lakes surveyed in the Gredos Massif (Central System, Spain). **Table S2.** Presence-absence of vascular hydrophytes in 17 softwater small lakes surveyed at the Gredos Massif (Central System, Spain).

**Appendix S1.** Full dataset.

**Appendix S2.** R coding.
