# Supplementary material for: Can environmental constraints determine random patterns of plant species co-occurrence?
Source: Ecol Evol. 2015 Feb 13;5(5):1088–99. doi: 10.1002/ece3.1349 (PMC4364823; doi:10.1002/ece3.1349)
Supplement: Supplementary file 1 [file ece30005-1088-sd1.doc]

**ONLINE SUPPLEMENTARY INFORMATION**

*Can environmental constraints determine random patterns of plant species co-occurrence?,* by Gonzalo García-Baquero & R. M. Crujeiras (e-mail address: [gonzalo.garcia-baquero@ehu.es](mailto:gonzalo.garcia-baquero@ehu.es))

**Table 1.** Location, physical characteristics and mid-summer chemical descriptors of *n* = 17 soft-water lakes surveyed in the Gredos Massif (Central System, Spain). Cartesian coordinates (*X*, *Y* in m) were derived from latitude and longitude data.

| Id. number | Lake name | Sector | X (m) | Y (m) | Elevation  (m) | Lake area  (m^2^) | Conductivity  (µS cm^-1^) | pH |
| --- | --- | --- | --- | --- | --- | --- | --- | --- |
| 1 | Negra | Bejar | 552 | 9165 | 2070 | 1450 | 13.0 | 6.0 |
| 2 | Duque | Bejar | 2479 | 9738 | 1595 | 203295 | 6.1 | 5.9 |
| 3 | Trampal 1 | Bejar | 1 | 10431 | 2125 | 6063 | 15.4 | 5.8 |
| 4 | Trampal 2 | Bejar | 386 | 10600 | 2115 | 15352 | 9.3 | 6.5 |
| 5 | Cuadrada | W Gredos | 9515 | 402 | 2085 | 7773 | 5.1 | 6.0 |
| 6 | Caballeros | W Gredos | 10588 | 1 | 2025 | 14027 | 9.1 | 6.3 |
| 7 | Barco | W Gredos | 9368 | 1550 | 1785 | 74781 | 7.2 | 6.1 |
| 8 | Nava | W Gredos | 11862 | 1239 | 1945 | 92268 | 5.6 | 6.1 |
| 9 | Grande | E Gredos | 37229 | 3198 | 1935 | 63076 | 4.8 | 6.2 |
| 10 | Gutre | E Gredos | 35171 | 4057 | 2300 | 960 | 8.7 | 6.2 |
| 11 | Cimera | E Gredos | 34845 | 4366 | 2140 | 44900 | 3.4 | 6.4 |
| 12 | Galana | E Gredos | 35046 | 4769 | 2135 | 15251 | 4.0 | 7.0 |
| 13 | Mediana | E Gredos | 35166 | 4916 | 2130 | 3240 | 4.5 | 6.5 |
| 14 | Brincalobitos | E Gredos | 35271 | 5095 | 2100 | 981 | 4.4 | 6.3 |
| 15 | Bajera | E Gredos | 35269 | 5211 | 2100 | 9599 | 3.9 | 6.9 |
| 16 | Majalaescoba | E Gredos | 34967 | 6309 | 1830 | 2615 | 6.9 | 6.6 |
| 17 | Lagunillas | E Gredos | 34282 | 6788 | 1915 | 5437 | 7.5 | 5.9 |

^a^ Lake area and elevation data were obtained from: Toro, M., I. Granados, et al. (2006). Limnetica **25** (1-2): 217-252.

**Table 2.** Presence-absence of vascular hydrophytes in 17 softwater small lakes surveyed at the Gredos Massif (Central System, Spain). Antinata, *Antinoria agrostidea* fma. *natans* (pseudo- hydrophyte); Callbrut, *Callitriche brutia* (batrachiid); Eleoacic, *Eleocharis acicularis* (amphiphyte); Isoeastu, *Isoetes velatum* subsp. *asturicense* (isoetid); Juncbulb, *Juncus bulbosus* var. *fluitans* (pseudo- hydrophyte); Myrialte, *Myriophyllum alterniflorum* (myriophyllid); Ranupelt, *Ranunculus peltatus* (batrachiid); Sparangu, *Sparganium angustifolium* (nymphaeid); Subuaqua, *Subularia aquatica* (isoetid). Identification numbers (Id.) as in Table 1.

| Id. | Antinata | Callbrut | Eleoacic | Isoeastu | Juncbulb | Myrialte | Ranupelt | Sparangu | Subuaqua |
| --- | --- | --- | --- | --- | --- | --- | --- | --- | --- |
| 1 | 1 | 1 | 0 | 1 | 0 | 0 | 1 | 1 | 0 |
| 2 | 1 | 1 | 0 | 1 | 0 | 0 | 1 | 0 | 0 |
| 3 | 0 | 1 | 1 | 1 | 1 | 0 | 1 | 1 | 1 |
| 4 | 0 | 1 | 0 | 1 | 0 | 0 | 0 | 1 | 1 |
| 5 | 1 | 0 | 0 | 0 | 0 | 0 | 0 | 0 | 0 |
| 6 | 1 | 1 | 0 | 1 | 1 | 0 | 1 | 1 | 0 |
| 7 | 1 | 0 | 0 | 1 | 0 | 0 | 1 | 0 | 0 |
| 8 | 0 | 0 | 0 | 0 | 0 | 0 | 0 | 0 | 0 |
| 9 | 1 | 1 | 0 | 1 | 1 | 1 | 1 | 1 | 0 |
| 10 | 0 | 0 | 0 | 0 | 1 | 0 | 0 | 0 | 0 |
| 11 | 0 | 0 | 0 | 0 | 0 | 0 | 0 | 0 | 0 |
| 12 | 0 | 0 | 0 | 0 | 0 | 0 | 0 | 1 | 0 |
| 13 | 0 | 0 | 0 | 0 | 0 | 0 | 0 | 1 | 0 |
| 14 | 0 | 0 | 0 | 0 | 0 | 0 | 0 | 0 | 0 |
| 15 | 0 | 0 | 0 | 0 | 0 | 0 | 0 | 1 | 0 |
| 16 | 0 | 1 | 0 | 0 | 0 | 0 | 1 | 0 | 0 |
| 17 | 1 | 0 | 0 | 1 | 0 | 0 | 0 | 0 | 0 |
